# Nucleic Acid–Driven Immunopathology in Sjögren’s Disease

**DOI:** 10.1155/jimr/9627427

**Published:** 2026-11-15

**Authors:** Yuanji Dong, Ting Wang, Huaxiang Wu

**Affiliations:** ^1^ Department of Rheumatology The Second Affiliated Hospital of Zhejiang University School of Medicine Hangzhou Zhejiang China z2hospital.com; ^2^ Department of Respiratory Disease Thoracic Disease Center The First Affiliated Hospital, College of Medicine Zhejiang University Hangzhou Zhejiang China zju.edu.cn

**Keywords:** immune cells, immunopathology, nucleic acid, salivary gland epithelial cells, Sjögren’s disease

## Abstract

Sjögren’s disease (SjD) is a chronic inflammatory disorder characterized by epithelial dysfunction and pathological lymphocyte homing. Clinically, it manifests with xerostomia, xerophthalmia, and fatigue and may involve systemic features such as arthralgia, cryoglobulinemia, interstitial lung disease, and peripheral neuropathy. Emerging evidence highlights the pivotal role of nucleic acids and their associated receptors in the pathogenesis of SjD. Aberrant nucleic acids can activate type I interferon (IFN‐I), the inflammasome, and proinflammatory signaling pathways. This cascade promotes the activation of immune cells and the release of cytokines, thereby exacerbating chronic inflammation. This review article elucidates the mechanisms of nucleic acid–mediated immunopathology in SjD and discusses potential therapeutic strategies.

## 1. Introduction

Sjögren’s disease (SjD), which was formerly known as Sjögren’s syndrome [1], is a prevalent chronic autoimmune disease characterized by sicca symptoms (xerostomia and xerophthalmia), fatigue, and chronic pain. Beyond exocrine gland dysfunction, SjD can involve multiple organ systems, with severe complications including mucosa‐associated lymphoid tissue (MALT) lymphoma and non‐Hodgkin lymphoma (NHL), contributing to elevated mortality rates compared to the general population [2–6]. As one of the most common rheumatic disorders, SjD exhibits wide variations in reported prevalence due to geographic, ethnic, and diagnostic criteria disparities [7–10]. To address this issue, a 2015 meta‐analysis estimated the global prevalence of SjD at 0.06% (95% CI 0.043%–0.078%) [11]. However, as the diagnostic criteria have been updated [12, 13], current evidence suggests that the overall prevalence of SjD ranges from 0.01% to 0.05% [14], with notably higher incidence rates observed in the UK (>0.1%) and China (0.05%–0.1%) [14].

The etiology of SjD remains elusive but involves genetic predisposition (e.g., HLA‐DQB1, interferon regulatory factor 5 [IRF5], and signal transducer and activator of transcription 4 [STAT4]), female sex bias (9:1–20:1 ratio), hormonal influences (e.g., postmenopausal estrogen decline), viral triggers (Epstein–Barr virus [EBV], cytomegalovirus [CMV], and human T‐cell leukemia virus type 1 [HTLV‐1]) [15–19]. Recent advances implicate salivary gland epithelial cells (SGECs) [20], immunofibroblasts [21], dysregulated T/B‐cell subsets [22–27], and innate immune cells (plasmacytoid dendritic cells [pDCs] and macrophages) in a cytokine network (type I interferon [IFN‐I], B cell activating factor [BAFF], interleukin‐6 [IL‐6], and C–X–C motif chemokine ligand 13 [CXCL13]) that drives tertiary lymphoid structure (TLS) formation and irreversible glandular damage [28–30]. A central theme emerging from these studies is the aberrant activation of IFN‐I, which is repeatedly implicated across different cell types and will be a focal point of this review.

Central to this immunopathology is the aberrant accumulation of endogenous nucleic acids (e.g., from cell death), which activate pattern recognition receptors (PRRs: including toll‐like receptors [TLRs], retinoic acid‐inducible gene I [RIG‐I]–like receptors [RLRs], cyclic GMP‐AMP synthase [cGAS], and absent in melanoma 2 [AIM2]). Under homeostatic conditions, self‐nucleic acids are sequestered or degraded to prevent autoimmunity; in SjD, however, impaired clearance and persistent signaling cascade create a vicious cycle of inflammation, cell death, and further nucleic acid release. This review article delineates the role of nucleic acid–sensing pathways in SjD immunopathology and explores emerging therapeutic strategies.

## 2. The Normal or Aberrant Nucleic Acids

Nucleic acids are essential biomolecules that encode genetic information and are broadly categorized into deoxyribonucleic acid (DNA) and ribonucleic acid (RNA). In humans, nuclear DNA (nDNA) serves as the primary genetic repository, while mitochondrial DNA (mtDNA) encodes components critical for energy metabolism. RNA exhibits remarkable diversity, including messenger RNA (mRNA), transfer RNA (tRNA), ribosomal RNA (rRNA), microRNA (miRNA), and long noncoding RNA (lncRNA), which collectively regulate gene expression and protein synthesis [31].

Under homeostatic conditions, self‐nucleic acids are largely sequestered within the nucleus, mitochondria, or endosomal compartments, and extracellular nucleic acids are rapidly cleared by nucleases and phagocytes. This compartmentalization and clearance are essential to prevent the inappropriate activation of nucleic acid–sensing pathways. In disease, such as in SjD, however, this balance is disrupted. Aberrant nucleic acids are defined here as DNA or RNA species that are mislocalized, chemically modified, inadequately cleared, or structurally abnormal, thereby gaining the capacity to activate host nucleic acid–sensing pathways and exert proinflammatory functions. Aberrant nucleic acids arise from several distinct biological processes (Table 1).

**Table 1:** Classification and sources of abnormal nucleic acids.

Category	Specific origins	Examples/mechanisms
Infection	DNA/RNA from bacteria, viruses, or fungi	Pathogen‐derived nucleic acids
Infection or stress	Cell death from infection or stress	Cellular debris releases nucleic acids
Epigenetic dysregulation	Transposable element activation	Inhibition of DNMTs leads to activation of ERVs, LINEs, SINEs, and aberrant dsRNA formation [32–36]
	Immunogenic dsRNA	IR‐Alus transcribed into ~300 bp dsRNA [35]
	Abnormal RNA modifications, splicing errors, and enzyme deficiencies	m6A modifications, splicing errors, or ADAR1 deficiency generating misfolded or unedited RNA [37–41]
Mitochondrial leakage	Cytosolic mtDNA and dsRNA accumulation	Via mPTP opening, MOMP, MDVs, or PNPase dysregulation [42, 43]
Nuclease/helicase deficiencies	Impaired degradation of free DNA or RNA	Deficiencies in DNAse I/II, DNAse IL3, TREX1, or SKIV2L activity [44–46]
Genomic instability	Excessive nuclear or mitochondrial DNA damage and leakage	nDNA or mtDNA damage and leakage into cytosol [47–51]
Other sources	Genetic mutations or synthetic nucleic acids	Direct introduction or mutation‐driven production [52]

Abbreviations: ADAR1, adenosine deaminase acting on RNA 1; DNMTs, DNA methyltransferases; ERV, endogenous retrovirus; LINEs, long terminal repeats; MDVs, mitochondrial‐derived vesicles; MOMP, mitochondrial outer membrane permeabilization; mPTP, mitochondrial permeability transition pore; nDNA, nuclear DNA; PNPase, polynucleotide phosphorylase; SINEs, short interspersed nuclear elements; SKIV2L, superkiller viralicidic activity 2‐like; TREX1, three‐prime repair exonuclease 1.

### 2.1. Infection or Stress Triggered

DNA/RNA released after microbial infection by bacteria, viruses, or fungi can directly serve as immunostimulatory nucleic acids, and infection or stress can also cause tissue or cellular damage, thereby releasing endogenous nucleic acids (Figure 1).

**Figure 1:**
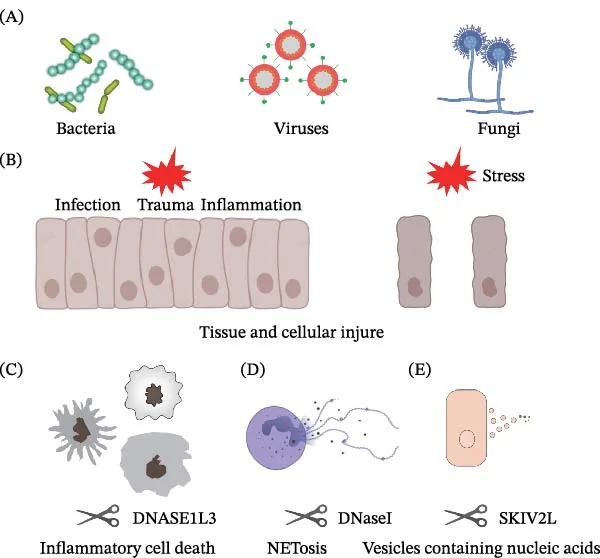
Major sources of aberrant extracellular nucleic acids. (A) Nucleic acids released by pathogenic microorganisms (bacteria, viruses, and fungi). (B) Nucleic acids are released due to tissue or cellular damage caused by infection, injury, inflammation, or stress. (C) Accumulation of extracellular nucleic acids resulting from deficiencies in nucleases DNASE1L3. (D) DNase I deficiency results in the accumulation of extracellular nucleic acids. (E) Helicase SKIV2L deficiency also leads to the accumulation of extracellular nucleic acids.

### 2.2. Epigenetic Dysregulation

Inhibition of DNA methyltransferases (DNMTs) can activate transposable element (TE), including endogenous retrovirus (ERV), long interspersed nuclear elements (LINEs), and short interspersed nuclear elements (SINEs), leading to the formation of aberrant double‐stranded RNA (dsRNA) [32–36]; inverted‐repeat Alu elements (IR‐Alus) transcribed into immunogenic dsRNA (~300 bp) [35]; RNA processing defects—including abnormal RNA modifications (e.g., m^6^A), splicing errors, and enzyme deficiencies such as adenosine deaminase acting on RNA 1 (ADAR1)—lead to the generation of misfolded or unedited RNA [37–41].

### 2.3. Mitochondrial Dysfunction

Cytosolic mtDNA and dsRNA accumulate due to mitochondrial permeability transition pore (mPTP) opening, mitochondrial outer membrane permeabilization (MOMP), mitochondrial‐derived vesicles (MDVs), or polynucleotide phosphorylase (PNPase) dysregulation [42, 43].

### 2.4. Nuclease or Helicase Deficiencies

The activity of DNase I/II, DNASE1L3, three‐prime repair exonuclease 1 (TREX1), or superkiller viralicidic activity 2‐like (SKIV2L) is impaired, which elevates the levels of free DNA or RNA [44–46].

### 2.5. Genomic Instability

Excessive nDNA or mtDNA damage and leakage [47–51] (Figure 2). Gene mutations and exogenous nucleic acids lead to the generation or introduction of aberrant nucleic acids [52]. While microbial nucleic acids trigger infection‐related inflammation, endogenous nucleic acids—particularly those arising from mitochondrial dysfunction or nuclease deficiency—drive autoimmunity by hyperactivating the pathways discussed below. Emerging evidence underscores the pivotal role of endogenous nucleic acids in autoinflammatory and autoimmune disorders.

**Figure 2:**
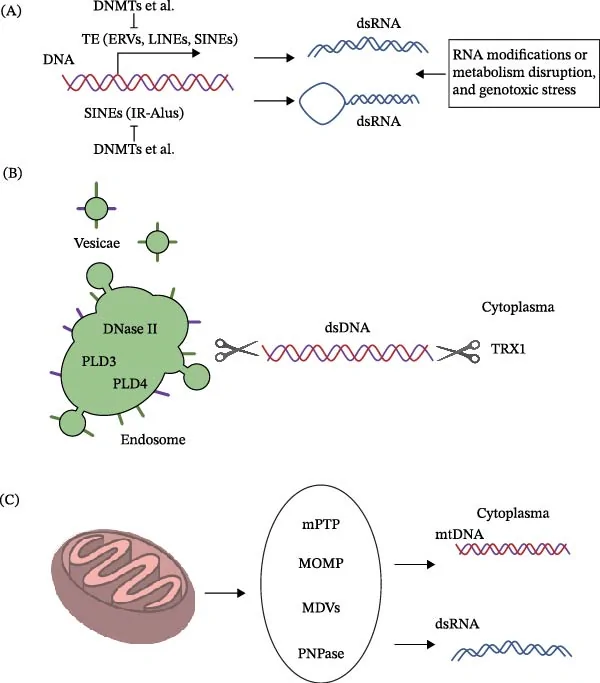
Major sources of aberrant intracellular nucleic acids. (A) Accumulation of abnormal dsRNA due to dysregulated transposable elements (ERVs, LINEs, and SINEs), RNA modification or metabolism disruption, and oxidative stress. (B) Defective clearance by nucleases (DNase II, PLD3, PLD4, and TREX1) leading to nucleic acid accumulation in endosomes and cytoplasm. (C) Mitochondrial dysfunction causing mtDNA/dsRNA release into the cytosol via mPTP opening, MOMP, MDVs, or PNPase dysregulation. dsRNA, double‐stranded RNA; ERV, endogenous retrovirus; LINEs, long interspersed nuclear elements; MDVs, mitochondrial‐derived vesicles; MOMP, mitochondrial outer membrane permeabilization; mPTP, mitochondrial permeability transition pore; mtDNA, mitochondrial DNA; PLD3, phospholipase D family member 3; PLD4, phospholipase D family member 4; PNPase, polynucleotide phosphorylase; SINEs, short interspersed nuclear elements; TREX1, three‐prime repair exonuclease 1.

## 3. Nucleic Acid Recognition Mechanisms

The host immune system employs sophisticated strategies to rapidly detect foreign nucleic acids derived from pathogenic microorganisms while implementing multiple safeguards to prevent inappropriate recognition of self‐nucleic acids. Nevertheless, under specific pathological conditions, these protective mechanisms may fail, enabling aberrant immune responses to endogenous nucleic acids. This section focuses on the recognition mechanisms of nucleic acids.

### 3.1. DNA Recognition Mechanisms

The following structures are capable of recognizing or binding DNA, including TLR9, cGAS, AIM2, Z‐DNA binding protein 1 (ZBP1), and interferon gamma‐inducible protein 16 (IFI16). Other sensors, such as DNA‐dependent protein kinase (DNA‐PK) and DEAD‐box helicase 41 (DDX41), are less understood in the context of SjD and will not be discussed in detail. TLR9 is an endosome/lysosome‐localized transmembrane protein that recognizes unmethylated CpG DNA motifs. Upon activation, TLR9 initiates a MyD88‐dependent signaling cascade, leading to nuclear factor kappa‐B (NF‐κB) transactivation and subsequent upregulation of proinflammatory cytokines (TNF‐α and IL‐6) [53]. cGAS is a cytosolic DNA sensor, and its catalytic core binds dsDNA in a sequence‐independent manner, requiring a minimum length of >45 bp to induce activation. Upon DNA binding, cGAS synthesizes the secondary messenger 2′3′ cyclic GMP‐AMP (2′3′‐cGAMP), which activates the stimulator of interferon genes (STING). This triggers two major pathways: the cGAS‐STING‐TANK binding kinase 1 (TBK1)‐IRF3 axis to produce IFN‐I and NF‐κB activation to release proinflammatory cytokines [54]. AIM2 is a cytoplasmic PRR of the pyrin and hematopoietic interferon‐inducible nuclear protein (HIN) domain family (PYHIN). It binds dsDNA (>80 bp, sequence‐independent) via its C‐terminal HIN‐200 domain and serves as a key inflammasome sensor. Upon DNA recognition, AIM2 recruits ASC and pro‐caspase‐1, leading to caspase‐1 activation and subsequent maturation of IL‐1β and IL‐18 [55]. IFI16 is a multifunctional nucleocytoplasmic shuttling protein that primarily localizes to the nucleus under resting conditions, where it interacts with chromatin. However, under viral infection, DNA damage, or autoimmune conditions, IFI16 translocates to the cytoplasm or mitochondria‐associated endoplasmic reticulum membranes (MERCs) to sense mtDNA. IFI16 is also a member of the PYHIN family, which contains two HIN‐200 domains (HIN‐A and HIN‐B) to bind dsDNA and an N‐terminal pyrin domain (PYD) to mediate inflammasome assembly. IFI16 can activate NOD‐like receptor family pyrin domain‐containing 3 (NLRP3) or AIM2 inflammasomes to secrete IL‐1β/IL‐18 and activate cGAS‐STING to produce IFN‐I [56]. ZBP1 is an innate immune receptor that specifically recognizes noncanonical Z‐DNA via its Zα domain. Upon binding, ZBP1 activates necroptosis, the inflammasome, and interferon pathways. ZBP1 plays a crucial role in antiviral defense and immune surveillance [57].

### 3.2. RNA Recognition Mechanisms

The mammalian immune system has evolved sophisticated mechanisms to detect RNA through specialized PRRs. These receptors can be categorized based on their RNA recognition specificity. TLR7 and TLR8 are mainly single‐stranded RNA (ssRNA) sensors. These endosomally localized transmembrane receptors recognize viral ssRNA and small RNAs [58, 59]. Both signal through the MyD88 adaptor, with TLR7 being predominantly expressed in pDCs and TLR8 primarily in myeloid cells [59]. The major dsRNA recognition sensors include TLR3, RIG‐I, melanoma differentiation‐associated protein 5 (MDA5), laboratory of genetics and physiology 2 (LGP2), NLRP1, protein kinase R (PKR), and 2′‐5′‐oligoadenylate synthetases (OASes) [60]. However, TLR3, RIG‐I, and MDA5 can also sense and recognize ssRNA, while TLR7 and TLR8 can bind to dsRNA [60–63]. This promiscuity may enhance viral RNA surveillance. TLR3, ubiquitously expressed endosomal receptor, preferentially binds long dsRNA (>40 bp) and signals through TIR‐domain‐containing adapter‐inducing interferon‐β (TRIF) [64–66]. RIG‐I recognizes short dsRNA (22–500 bp) and ssRNA (optimal activity at 50–300 nt), requiring 5′‐ppp termini and a panhandle structure [60, 61]. MDA5 detects long dsRNA (>500−1000 bp; length‐dependent activation) and ssRNA (>1 kb) independent of 5′‐ppp [60, 61]. LGP2 lacks N‐terminal caspase activation and recruitment domain (CARD) domains and exhibits complex regulatory roles [67–71]. RIG‐I, MDA5, and LGP2 share conserved DExD/H helicase domains, with RIG‐I and MDA5 containing additional N‐terminal CARD domains that mediate mitochondrial antiviral signaling protein (MAVS) interaction, ultimately activating IRF‐3/7 and NF‐κB [68]. NLRP1, as an inflammasome, directly detects long dsRNAs (>500 bp), triggering IL‐1β/IL‐18 releases and gasdermin D (GSDMD)–mediated pore formation in a length‐dependent manner [72, 73]. PKR, as a dsRNA‐dependent kinase (activated by >30 bp dsRNA), dimerizes and autophosphorylates upon binding, subsequently phosphorylating eIF2α to inhibit translation. PKR enhances IFN‐α/β production through mRNA stabilization rather than transcriptional upregulation [74, 75]. OASes, as enzymes, catalyze 2′‐5′ oligoadenylate (2‐5A) synthesis upon dsRNA binding, activating RNase L to degrade RNA and generate fragments that can further stimulate RLRs or MAVS‐dependent NLRP3 inflammasomes [76–78].

## 4. Nucleic Acid–Mediated Immune Disorders and Inflammation in SjD

### 4.1. Abnormal Nucleic Acid and Activation of the IFN‐I in SjD

Emerging evidence demonstrates significantly elevated levels of aberrant nucleic acids in the serum and salivary glands of SjD. Histological evidence shows DNA deposits in SGECs and infiltrating macrophages [79, 80]. Peripheral blood mononuclear cells (PBMCs) from SjD exhibit increased short‐fragment cytoplasmic DNA [79], all of which implicates dysregulated nucleic acid sensing in disease pathogenesis [79, 81, 82]. Multiple sources contribute to the nucleic acid accumulation, including virus infection (e.g., EBV and HTLV‐1) [83, 84]; LINE1 hypomethylation [85, 86]; mitochondrial dysfunction [87–89]; cell death processes such as pyroptosis, necroptosis, ferroptosis, and neutrophil extracellular trap (NETosis) [90, 91]; and nucleic acid‐containing exosomes [92]. However, the key mechanistic question concerns not merely the accumulation of aberrant nucleic acids but rather its relationship with the well‐established IFN‐I signature in SjD.

SjD exhibits significant IFN‐I characteristics [93, 94]. In studies utilizing gene expression microarray data to identify potential genomic biomarkers for SjD, 13 (such as interferon‐induced protein 44‐like [IFI44L], myxovirus resistance protein 1 [MX1], epithelial stromal interaction 1 [EPSTI1], 2′‐5′‐oligoadenylate synthetase 1 [OAS1], etc.) out of the 19 genes identified as critical for accurate disease classification were found to be interferon‐related genes [95]. In addition, the expression of interferon‐induced protein myxovirus resistance protein A (MxA) is elevated in patients with active SjD [96]. Although aberrant nucleic acids are detected by distinct cytosolic sensors, these pathways converge on the phosphorylation of IRF3/IRF7 and the subsequent production of IFN‐I. IFN‐I enhances dendritic cell (DC) maturation, MHC class II expression, and autoantigen presentation; promotes B‐cell activation and plasma cell differentiation; and induces BAFF and CXCL13 expression while supporting TLS formation. This chronic inflammation further promotes IFN‐I production by both pDCs and SGECs, thereby establishing a self‐perpetuating cycle in which nucleic acid release activates IFN‐I signaling, and IFN‐I amplifies immune cell activation and further promotes nucleic acid and IFN‐I release, ultimately leading to tissue damage and disease progression. Consistent with this, patients with high interferon gene signature (IGS) scores have more prominent sicca symptoms [97]. Yet paradoxically, the overall disease burden does not increase compared with low‐IGS patients [97]. These findings position abnormal nucleic acid accumulation and consequent IFN‐I activation as central players in SjD; however, several key questions remain unresolved. First, it is still unclear whether aberrant nucleic acid accumulation is a primary driver or a secondary consequence of chronic inflammation. Second, most evidence linking specific sensing pathways to IFN‐I production comes from cell lines or mouse models, and direct validation in patient‐derived B cells or salivary gland tissue is lacking. Third, the paradoxical observation that high‐IGS patients do not have an increased overall disease burden suggests that IFN‐I may drive specific clinical features rather than global disease severity. Future studies using patient‐derived B cells, humanized mouse models, and longitudinal cohorts, together with pathway‐specific inhibitors, are needed to establish causality and to determine which nucleic acid–sensing pathways are most relevant to SjD pathogenesis.

### 4.2. The Role of Nucleic Acid Sensing in the SGECs and Immune Cells

In SjD, SGECs are not merely structural components but active contributors to disease pathogenesis [20, 98]. On the one hand, SGECs express various immune‐related molecules, including CD40, B7, MHC‐II, ICAM‐1, and multiple TLRs, thereby acquiring nonclassical antigen‐presenting cell (APC) functions and the ability to recognize pathogen‐associated molecular patterns (PAMPs) and damage‐associated molecular patterns (DAMPs) [98]. On the other hand, they produce and secrete numerous cytokines and chemokines, such as IL‐33, IL‐6, BAFF, IL‐7, and CXCL10, which in turn regulate the survival and differentiation of T cells (Th1 and Tfh) and B cells (FcRL4^+^Tbet^+^CD11c^+^) [26, 99–106]. Concurrently, SjD is also characterized by abnormal lymphocyte homing and the infiltration and activation of various immune cells [107]. In addition to aggregating around ducts and impairing glandular function, lymphocytes can form specialized structures such as lymphoepithelial lesions (LELs) and TLSs [28, 106]. Furthermore, monocytes/macrophages (secreting IL‐6 and IL‐1β) [108], pDCs (secreting IFN‐α) [109], and NK cells (secreting IFN‐γ) are also extensively involved in the pathological process of SjD [110]. Against this background, this section focuses on integrating the nucleic acid–sensing mechanisms in SGECs (Figure 3) and immune cells (Figure 4), with particular emphasis on key signaling pathways that have been functionally validated.

**Figure 3:**
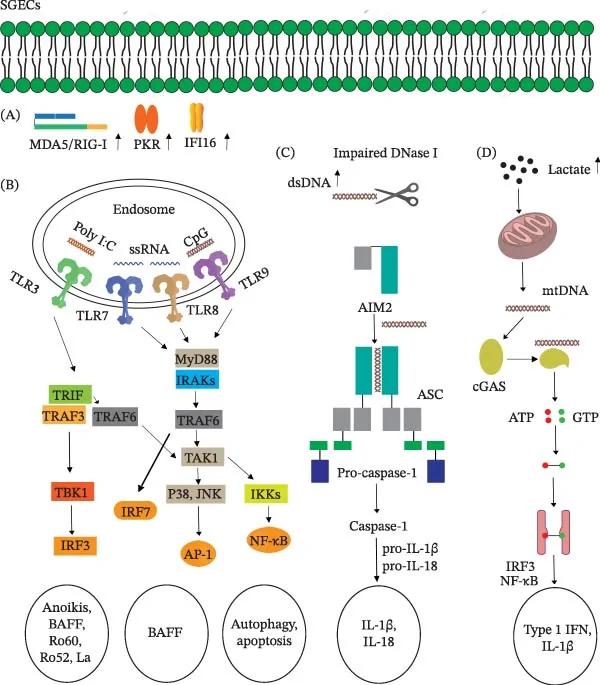
Mechanisms of nucleic acid sensing in SGECs in SjD. (A) Upregulated expression of MDA5, RIG‐I, PKR, and IFI16 in SGECs of SjD patients. (B) Aberrant activation of TLR3, TLR7, TLR8, and TLR9 pathways in SGECs of SjD, leading to BAFF secretion, autoantigen release, and induction of apoptosis, autophagy, and anoikis. (C) Impaired DNase I activity in SGECs of SjD results in abnormal accumulation of dsDNA and subsequent AIM2 inflammasome activation. (D) Lactate accumulation in SGECs of SjD causes mitochondrial dysfunction, releasing mtDNA that activates the cGAS pathway, promoting IFN‐I and IL‐1β production. AIM2, absent in melanoma 2; BAFF, B cell activating factor; cGAS, cyclic GMP‐AMP synthase; IFI16, interferon gamma‐inducible protein 16; IFN‐I, type I interferon; MDA5, melanoma differentiation‐associated protein 5; PKR, protein kinase R; RIG‐I, retinoic acid‐inducible gene I; SGECs, salivary gland epithelial cells; SjD, Sjögren’s disease; TLR, toll‐like receptor.

**Figure 4:**
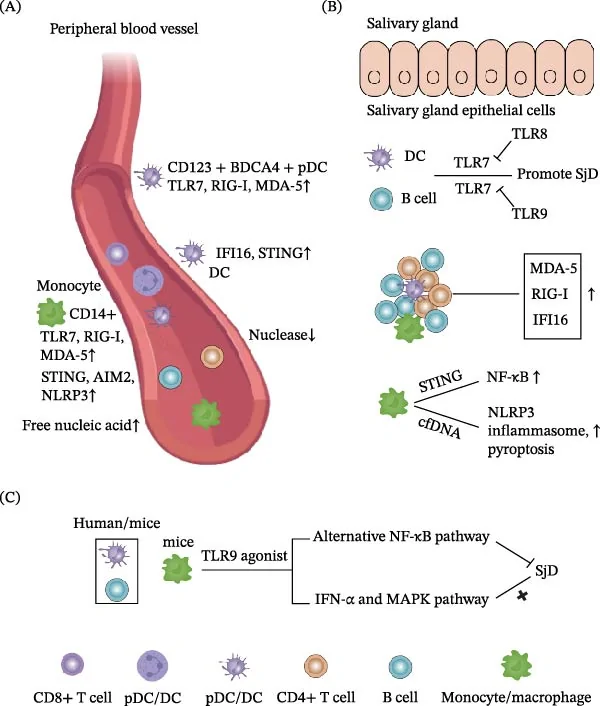
Mechanisms of nucleic acid sensing in immune cells in SjD. (A) Reduced nuclease activity and increased circulating cell‐free nucleic acids in peripheral blood of SjD; increased levels of IFI16 and STING in DCs of PBMC in SjD; upregulated TLR7, RIG‐I, and MDA5 in CD123^+^ BDCA2^+^ pDCs of PBMC in SjD; and elevated TLR7, RIG‐I, MDA5, STING, AIM2, and NLRP3 in CD14^+^ monocytes of PBMC in SjD. (B) Overexpression of MDA5, RIG‐I, and IFI16 in infiltrating immune cells of SjD; promoting disease progression via hyperactivated TLR7 signaling in DCs and B cells. (C) TLR9 dual roles in SjD: protective effect when activating the alternative NF‐κB pathway; pathogenic effect when triggering IFN‐α or MAPK pathways. BDCA2, blood dendritic cell antigen 2; DCs, dendritic cells; ISGs, interferon‐stimulated genes; MAPK, mitogen‐activated protein kinase; NLRP3, NOD‐like receptor family pyrin domain‐containing 3; PBMCs, peripheral blood mononuclear cells; pDCs, plasmacytoid dendritic cells; SjD, Sjögren’s disease; STING, stimulator of interferon genes.

#### 4.2.1. RNA Sensing Pathways (TLR3, TLR7, TLR8, RIG‐I, and MDA5)

Studies have shown that the activation of TLR3 on SGECs by polyI: C (dsRNA) promotes aberrant expression and altered localization of SjD‐associated autoantigens (Ro60, Ro52, and La), which are subsequently recognized by the immune system via exosomes [111]. Moreover, polyI: C induces anoikis in SGECs [112] and triggers BAFF secretion through a PKR‐dependent mechanism [113, 114]. Similarly, ssRNA (e.g., Sendai virus) induces BAFF production via the TLR7/TLR8 pathway [114]. In immune cells, multiple TLRs may play roles in SjD, among which the nucleic acid–sensing TLR3, TLR7, and TLR8 have particularly complex and context‐dependent functions [115, 116]. In the submandibular glands (SMGs) of C57BL/6.In NOD‐Aec1Aec2 mice (a SjD model), transcriptomic analysis revealed the upregulation of TLR3 and TLR7, along with their downstream signaling molecules, suggesting early activation of TLR3 and TLR7 in the disease [117]. Furthermore, TLR8‐deficient C57BL/6 mice spontaneously develop a SjD‐like phenotype due to enhanced DC‐mediated TLR7 signaling [118]. Consistent with these animal data, patients with SjD exhibit significantly increased expression and signaling activity of TLR7 in PBMCs, pDCs, and infiltrating monocytes, while the role of TLR8 is relatively minor, indicating that TLR7 is a key node in immune cell‐driven SjD pathology [119–122]. Meanwhile, multiple interferon‐stimulated genes (ISGs) involved in cytoplasmic RNA‐sensing pathways are significantly upregulated in SjD, with RIG‐I and MDA5 staining predominantly observed in infiltrating immune cells [122, 123]. Correspondingly, in peripheral blood from SjD patients with a prominent interferon signature, cytoplasmic TLR7, RIG‐I, and MDA5 are markedly upregulated in pDCs and monocytes [122]. Under physiological conditions, SGECs express only low levels of RIG‐I, MDA5, and PKR [122], but these molecules can be induced to higher expression levels in SjD [124]. Although the RLR‐MAVS and PKR pathways are functionally active in primary human SGECs, their precise pathological roles in SjD remain unclear [124, 125]. Collectively, these findings point to a synergistic interaction between TLRs and RLRs, suggesting that the TLR–RLR regulatory network may serve as a promising target for blocking interferon‐related pathways. Nevertheless, the therapeutic potential of targeting this network remains to be validated in preclinical models and clinical studies.

#### 4.2.2. DNA Sensing Pathways (AIM2, cGAS‐STING, IFI16, and TLR9)

In SGECs, impaired DNase I activity leads to elevated cytosolic DNA levels, resulting in autonomous AIM2 inflammasome activation and persistent chronic inflammation [80]. Accumulation of lactate in SjD compromises mitochondrial integrity in SGECs, causing mtDNA leakage and activation of the cGAS‐STING pathway [126]. Notably, not only SGECs but also lacrimal gland myoepithelial cells can be directly activated by genomic DNA (gDNA) to produce AIM2, thereby promoting the secretion of IL‐1β and IL‐18; concurrently, gDNA can activate STING and promote IFN‐β secretion, further enhancing IL‐1β release [127]. In SjD, IFI16 is aberrantly expressed not only in infiltrating immune cells within salivary glands [128, 129] and in circulating pDCs [122, 130–132] but also in SGECs [133], where transfection with dsDNA induces its oligomerization and release upon CTL‐ and NK‐mediated membrane damage, leading to the production of anti‐IFI16 autoantibodies that correlate with more severe clinical manifestations [134]. Tissue‐infiltrated monocytes exhibit high STING expression, while peripheral blood monocytes and pDCs display hyperresponsiveness to STING stimulation [135]. Of particular note, the administration of a STING agonist alone is sufficient to induce a SjD‐like phenotype in mice [136]. Furthermore, macrophages from SjD patients show cytoplasmic DNA deposition, accompanied by NLRP3 inflammasome activation and pyroptosis; moreover, free nucleic acids isolated from the serum of SjD patients can effectively activate the NLRP3 and AIM2 inflammasomes in monocytes [79]. These findings indicate that the cGAS‐STING and AIM2 pathways are aberrantly activated in both SGECs and immune cells.

Regarding the role of TLR9 in SjD, at the cellular level, CpG DNA can induce autophagy and apoptosis in SGECs via the p38 and JNK signaling pathways by stimulating TLR9 [137]. Consistent with this mechanism, an increased proportion of TLR9/p38 mitogen‐activated protein kinase (MAPK) double‐positive cells in PBMCs of female NOD mice (5–10 weeks old) correlates with reduced salivary secretion, and similar TLR9/p38 MAPK double‐positive lymphocytes are also detected in the SMG [138]. Animal studies have shown that distinct TLR9 signaling pathways exert differential regulatory effects on the disease: Activation of the alternative NF‐κB pathway ameliorates SjD‐like symptoms in NOD mice, whereas activation of the IFN‐α or MAPK pathways exacerbates disease manifestations [139]. Notably, TLR9 deficiency leads to more severe sialadenitis in MRL/lpr mice, and given that TLR9 negatively regulates TLR7, it is hypothesized that this phenomenon may result from excessive TLR7 signaling in B cells [118, 140].

In addition, NETosis is significantly enhanced in SjD [90]. Given that NETosis releases chromatin into the extracellular space and that DNase I function is impaired in SjD, it is plausible that NETosis plays an important role in nucleic acid–mediated immune inflammation, although this hypothesis requires further validation [90, 91]. Furthermore, SGECs can undergo proinflammatory cell death, such as pyroptosis and ferroptosis, which further exacerbate the release of self‐nucleic acids and amplifies nucleic acid–mediated immune activation [91].

In summary, both SGECs and immune cells have been confirmed to act as active drivers of inflammatory responses through their intrinsic nucleic acid sensors. Among these, SGECs particularly rely on the AIM2 and cGAS‐STING pathways, while TLR3/TLR7 are mainly involved in BAFF secretion and autoantigen expression. In immune cells, both RNA‐sensing pathways (TLR7 and RIG‐I/MDA5) and DNA‐sensing pathways (IFI16, STING, and AIM2) exhibit hyperactivation, with TLR7 playing a particularly dominant role in B cells and pDCs. In contrast, the role of TLR9 is relatively minor, and it may indirectly influence disease progression through the negative regulation of TLR7. These findings further suggest that dysregulated nucleic acid sensing may serve as a link between epithelial cell activation and immune cell dysfunction. Although the specific mechanisms differ, these processes collectively contribute to the IFN‐I signature in SjD and provide a foundation for the persistence of chronic inflammation.

### 4.3. From Nucleic Acid Sensing to Hallmark Autoantibodies: Anti‐Ro/SSA and Anti‐La/SSB

A critical but relatively underexplored question is how nucleic acid–mediated innate immune activation contributes to the generation of hallmark SjD autoantibodies, particularly anti‐Ro/SSA and anti‐La/SSB. These autoantibodies are central to disease classification, diagnosis, and pathogenesis, yet the mechanisms linking nucleic acid sensing to their production remain incompletely defined. Several lines of evidence suggest a mechanistic continuum from nucleic acid release to loss of B‐cell tolerance.

To generate autoantibodies, the process begins with the exposure of Ro60, Ro52, and La [82, 111]. Subsequently, the formation of RNA–autoantigen complexes activates endosomal TLR7/TLR8 and cytosolic RIG‐I/MDA5, promoting IFN‐I production and proinflammatory cytokine release. IFN‐I, in turn, promotes DC maturation, MHC class II expression, and autoantigen presentation, thereby facilitating the activation of autoreactive T cells and B cells. Ultimately, within the complex immune microenvironment, long‐lived plasma cells that produce autoantibodies are generated. These autoantibodies can further form immune complexes with RNA, which may create a self‐reinforcing loop that links nucleic acid sensing to adaptive autoimmunity. However, the specific mechanisms involved remain unclear and require extensive further investigation.

## 5. Potential Therapeutic Targets

### 5.1. TLR Inhibitors

Given the central role of TLRs in SjD pathogenesis [116], TLR inhibition represents a logical therapeutic strategy. Hydroxychloroquine, a clinically widely used TLR inhibitor (primarily suppressing TLR9 with weaker effects on TLR7/8), failed to demonstrate significant improvement in systemic inflammation or sicca symptoms in two Phase III clinical trials for SjD [141, 142]. However, hydroxychloroquine combined with leflunomide showed efficacy in slowing disease progression in a randomized double‐blind trial [143]. Multiple novel approaches are under development: M5049 (Enpatoran) blocks endogenous RNA ligands while inhibiting TLR7/8 signaling [144]; IRS‐954 competitively binds TLR7/9 via specific DNA sequences [145]; immunomodulatory oligonucleotides contain synthetic dinucleotides and novel DNA structures to simultaneously inhibit TLR7/8/9 [146]; 7f [147], BMS‐986256 (Afimetoran) [148], KBD4466 [149], and TH 407b [150], as small molecule inhibitors, suppress TLRs; DS‐7011a [151], as monoclonal antibody, inhibits TLR7. While most evidence derives from studies of SLE, the application in SjD still requires validation. Considering the key role of TLR7, it is necessary to focus on the therapeutic potential of TLR7 inhibitors while also paying attention to possible side effects.

### 5.2. Inflammasome Inhibitors

Given the autonomous AIM2 inflammasome activation in SGECs [80] and the AIM2/NLRP3 activation in macrophages, targeting inflammasomes is another promising avenue. Thalidomide is a potent anti‐inflammatory and antiangiogenic agent that may suppress the assembly of the AIM2 inflammasome [152]. 4‐Sulfonic calixarenes and suramin can reversibly bind the HIN domain of AIM2, blocking dsDNA‐driven inflammasome assembly [153]. Preclinical studies have demonstrated that pharmacological intervention (iguratimod, grp78, and ruscogenin) targeting NLRP3 alleviates SjD symptoms [154–156]. However, clinical evidence supporting these therapeutic approaches in SjD remains lacking.

### 5.3. Restoring Nuclease Activity and the Inhibition of cGAS‐STING

Emerging evidence demonstrates significant impairment of nucleic acid clearance mechanisms in SjD. DNase I activity decreased in SGECs [80]. DNase II function is diminished in PBMCs [79]. These deficiencies correlate with abnormal accumulation of short‐fragment cytoplasmic DNA, which potently activates inflammatory signaling and drives disease progression. The OAS‐RNase L system, which normally cleaves viral and self‐RNA through 2′‐5′‐oligoadenylate production [77, 78, 157], shows compromised function in SjD. OAS1 variants lead to inefficient viral clearance and persistent interferon production, both of which are associated with SjD [158]. While restoring nuclease activity represents a logical therapeutic approach to reduce aberrant nucleic acid accumulation and subsequent inflammation, current strategies remain theoretical without clinical validation. As noted in Section 3.2, cGAS‐STING activation is sufficient to induce SjD‐like phenotypes in mice [136], making it a high‐priority target. It is reported that rapamycin can negatively regulate the cGAS‐STING pathway by activating autophagy, thereby improving the pathology of SjD [159]. Multiple targeting cGAS‐STING pathway inhibitors have been developed (reviewed in [160]). However, their application in SjD remains very limited. We look forward to more studies in the future to explore their therapeutic potential in SjD.

### 5.4. IFN‐I and JAK Inhibitors

As repeatedly emphasized, IFN‐I is a key downstream effector of nucleic acid sensing in SjD. Transcriptomic analysis of salivary gland tissue [161] and SGECs [104] of SjD revealed that ISGs were upregulated. In SjD, pDCs secrete a large amount of IFN‐α, and SGECs can also produce IFN‐α [162]. Persistent activation of IFN‐I is believed to promote and maintain chronic inflammation of SjD, leading to tissue damage and disease progression. Anifrolumab, a monoclonal antibody against the IFN‐α/β receptor (IFNAR), has shown efficacy in SLE [163]. Currently, a phase II trial (ANISE‐II, NCT05383677) is evaluating the therapeutic effect of Anifrolumab in SjD. A variety of cytokines transmit downstream signals via JAK‐STATs, including interferon. Therefore, JAK is also a potential target for SjD. It is reported that tofacitinib can improve the symptoms of SjD [164]. Tofacitinib can modulate IFN signaling and suppress Tfh and Tph, which contribute to B‐cell hyperactivity and autoantibody production [165]. These results indicate that targeting IFN‐I and JAKs represents a promising therapeutic avenue in SjD. While Anifrolumab and JAK inhibitors are under investigation, further clinical validation is needed to establish their efficacy, safety, and optimal use in SjD management.

## 6. Conclusions

SjD is a chronic autoimmune disorder characterized by epithelial cell damage and aberrant lymphocyte homing. This review has systematically examined the roles of nucleic acid–sensing pathways in SjD, with particular focus on five key themes: (1) epithelial cell‐intrinsic AIM2 inflammasome activation, (2) hyperactive TLR7 signaling in B cells and pDCs, (3) synergistic crosstalk between TLRs and RLRs, (4) aberrant cGAS‐STING pathway activation in both epithelial and immune cells, and (5) the unifying downstream role of IFN‐I. While these pathways are discussed in distinct sections, they are interconnected in vivo, and their relative contributions may vary across patients and disease stages.

While targeting abnormal nucleic acid signaling offers promising therapeutic options, we also notice that many treatments are still in the early stages, and clinical data remain very limited. In addition, high disease heterogeneity, complex feedback mechanisms within nucleic acid–sensing networks, and potential off‐target effects all exacerbate the complexity. Future research priorities: (1) mechanistic investigations (decipher epigenetic regulation of nucleic acid sensors, elucidate tissue‐specific signaling variations, and characterize sex‐dimorphic aspects of nucleic acid sensing), (2) biomarker development (identify predictive signatures for treatment response, validate noninvasive monitoring tools, and establish pathway‐specific biomarkers), (3) preclinical models (utilize patient‐derived organoids for drug screening, establish humanized mouse models incorporating patient immune cells, and develop microphysiological systems mimicking glandular microenvironments), and (4) therapeutic optimization (explore combination strategies, develop cell‐type selective modulators, and test sequential treatment algorithms). These efforts will help promote the development of precision medicine for SjD and ultimately improve the clinical prognosis.

## Author Contributions

All authors were involved in the writing and critical review of this article.

## Funding

This work was supported by a grant from the National Natural Science Foundation of China (Grant 82402087).

## Disclosure

All authors have approved the final version.

## Conflicts of Interest

The authors declare no conflicts of interest.

## Data Availability

Data sharing is not applicable to this article as no datasets were generated or analyzed during the current study.
